# Professional Quality of Life, Engagement, and Self-Care in Healthcare Professionals in Ecuador during the COVID-19 Pandemic

**DOI:** 10.3390/healthcare9050515

**Published:** 2021-04-29

**Authors:** María Elena Cuartero-Castañer, Paula Hidalgo-Andrade, Ana J. Cañas-Lerma

**Affiliations:** 1 Philosophy and Social Work Department, Universitat de les Illes Balears, 07122 Palma, Spain; me.cuartero@uib.es (M.E.C.-C.); ana.lerma@uib.es (A.J.C.-L.); 2 School of Psychology, Universidad de Las Américas, Quito 17041, Ecuador

**Keywords:** COVID, engagement, self-care, professional quality of life, Ecuador, healthcare professionals

## Abstract

The COVID-19 pandemic has highlighted the importance of healthcare workers and their professional quality of life. This quantitative cross-sectional study aims at exploring the professional quality of life, work engagement, and self-care of healthcare workers during the COVID-19 pandemic in Ecuador. A convenience sample of 117 participants completed an online voluntary and anonymous survey between April and July 2020. It contained a sociodemographic section, the Professional Quality of Life questionnaire V, the work engagement scale, and the scale of self-care behaviors for clinical psychologists. Results show that healthcare workers have an average quality of life with high levels of compassion satisfaction and average levels of compassion fatigue and burnout. Data also indicate that the sample frequently engaged in self-care practices and had high levels of work engagement. The regression analyses reveal that gender, the number of patients per week, the perceived fairness of the salary, among other variables are possible predictors of professional quality of life, frequency of self-care practices, and engagement. This study contributes to the understanding of these variables among healthcare professionals in Ecuador. These results should be considered when planning policies and prevention intervention efforts to promote professionals’ wellbeing.

## 1. Introduction

As in many other countries, Ecuador has exponentially increased life expectancy and aging in the last 30 years [1]. This demographic change entails a significant increase in social and medical needs. In recent decades, the bio-medical model has dominated the health field due to its technological and scientific advances that have managed to find cures or treatments for many diseases. However, most current trends advocate a humanization of care that offers a bio-psycho-socio-spiritual perspective that influences patients’ quality of care and their families [2]. Thus, recently, subtle attention has been paid to professionals who provide such care.

The latest events of the COVID-19 pandemic have highlighted that the health system is supported by a great network of professionals. Their involvement has gone beyond professional goals, fully immersing them in an extreme emotional maelstrom [3]. Ecuador is among the worst-hit countries in the world [4]. On April 2021, Ecuador reported 12,106 confirmed deaths and 4898 probable deaths from COVID-19, of which Pichincha, the province where the capital city of Quito is located, is the most affected region in the entire country with 37.7% of accumulated confirmed cases [5], and other studies show that there have been many more unregistered deaths [6].

In situations such as the one we currently experience, besides specific knowledge, healthcare workers require good professional quality of life, personal and professional self-care practices, listening skills, as well as emotion and stress management to provide quality care. It is crucial that they also feel cared for by their work and social environments, which help maintain an adequate emotional balance to face adverse conditions [7].

In moments of distress, professionals’ empathic care shows respect, recognition, legitimation, listening, and appreciation for patients. This attitude brings proximity and eases discomfort, enabling a better bond between professionals and patients [8]. In 2018, Howick et al. published a systematic review and meta-analysis on the benefits of close and quality care by healthcare personnel. Twenty-eight articles demonstrate that health professionals who express empathy, proximity, and security, as well as those who transmit positive messages, bring improvements in their patient’s psychological and physical conditions and general satisfaction [9]. Caregiving experiences can be rewarding and overwhelming at the same time and may influence the ability to provide safe and quality healthcare [10]. Thus, despite being competent professionals, their experiences during a global pandemic can influence their quality of life and work engagement.

The Professional Quality of Life is a complex concept associated with the work environment (organizational and task performed), personal characteristics, and the relationship between the professional and a traumatic event experienced directly or indirectly [11]. It includes adverse effects of caring for others, such as Burnout (BO) and Compassion Fatigue (CF), as well as positive results such as Compassion Satisfaction (CS) [12].

BO is a three-dimensional syndrome characterized by emotional exhaustion, depersonalization, and low professional fulfillment [13] that can occur in people who work in contact with clients or patients. It is a progressive loss of idealism, energy, and goals due to work stress [14]. Despite the different conceptions about BO’s etiology and maintenance, there are common characteristics [15]. For example, its symptoms are related to work and are associated with physical and emotional exhaustion, and depression, it may occur in people without previous pathologies, atypical symptoms appear due to physical distress, and there is a reduction in work effectiveness and performance due to negative attitudes and behaviors.

CF is a general term applied to anyone who suffers due to being a formal caregiver in health, psychology, and social fields and refers to the natural emotional behaviors and reactions derived from knowing about a significant traumatic event [16]. CF is also conceptualized as a state of exhaustion and biological, psychological, and social dysfunction resulting from prolonged compassion stress [17]. If professionals experience such a phenomenon, their ability to empathize, connect, and help their clients could be seriously diminished. CF affects the behavioral, affective, somatic, interpersonal, and cognitive aspects of a person [18].

The most crucial difference between BO and CF is the work environment’s influence [19]. While the CF is based on the relationship between the professional and the client through therapeutic alliance and empathy, BO can incorporate other variables outside of this relationship. If CF is unnoticed, it can lead to severe symptoms of secondary traumatization or BO and, consequently, to the consumption of anxiety-related medication, absenteeism, and work abandonment. Moreover, it could affect the quality of the provided services, the patients, and their environment.

On the other hand, CS acts as a buffer, in contrast to CF, to the risk of suffering BO or secondary traumatic stress [20]. CS leads to situations in which professionals indirectly benefit from their patients’ evolution [21], personal growth, and therapeutic gains, given that achievements, joy, and satisfaction are also shared within the patient–carer relationship. CS is defined as the ability to receive gratification from providing care [22]. The latest research shows that CS is related to self-efficacy at work and more adequate coping mechanisms [23]. In addition, physicians who work from an integrative model present higher CS levels than those who work from a biomedical approach [24].

Work engagement is a positive mental state linked to work activity [15]. It has three components: vigor, absorption, and dedication. Vigor is the high level of energy and readiness for mental recovery during work; the ability to be resilient in the face of adversity and invest effort and perseverance in their work. Absorption is understood as concentration and control of the situation, and the feeling of enjoyment with the job done. Dedication is the high sense of significance with the role carried out in the organization and the pride and inspiration generated when doing it [15]. The positive effect of high engagement supposes an increase in a person’s motivations for their work by activating their internal resources to achieve the proposed objectives [25]. Furthermore, health and motivation can affect and change the work environment, highlighting the dynamic nature of the relationships between the work environment and wellbeing.

As a phenomenon inherent to the help process and complex work environment, both CF and BO are sometimes difficult to prevent. To minimize their effects and increase CS and engagement, it is necessary to plan and develop conscious actions. Self-care are activities carried out by individuals, families, or communities, intending to promote health, prevent and limit disease, bearing in mind an institutional, personal, and community dimension [26]. In recent years, there has been a growing interest in the role of self-care in professionals who work in environments with high levels of stress. 

Self-care is a responsibility that begins during educational training and continues as a lifestyle [27]. The combination of personal and professional self-care strategies is essential and includes an intentional engagement or involvement in global practices of health and wellbeing of oneself personally and applied to the professional role [28]. 

Recent literature suggests that the absence or inconsistency of self-care correlates with a higher risk of CF, secondary traumatic stress, BO [29], and inadequate engagement with clients [30]. Individual commitment to self-care, coupled with healthcare organizations’ support, creates the optimal framework in which BO can be mitigated [31]. The latest research highlights the importance of self-care and links it to professional competence, ethics, prevention, management of work stress, survival, and growth of the profession [32].

### The Current Study 

Initially, this study was designed at the end of 2019 to examine the quality of life of palliative care health personnel in Quito, Ecuador. Due to the unexpected arrival of COVID-19, which revolutionized the health and social landscape of the entire world, it was considered necessary to carry out a more specific study adapted to the situation.

A recent Spanish study shows that during the COVID-19 pandemic, health professionals showed high levels of CF and BO, which had an impact on their mental health and professional quality of life [33]. The study insists on implementing long-term contingency programs aimed at improving the emotional wellbeing of healthcare professionals. Another research from Italy indicates that higher levels of BO and CF and lower levels of CS were present in professionals who worked in areas with higher rates of COVID-19 and highlight the need for psychological support [34]. Given these antecedents in Europe, this research aims to study the relationships and predictors of professional quality of life, engagement, and self-care of healthcare professionals in Ecuador (Quito, South America) during the COVID-19 pandemic.

## 2. Materials and Methods

This quantitative cross-sectional design study is part of an international cooperation project between the University of the Balearic Islands in Spain and Universidad de Las Américas in Ecuador. This article’s data were collected between April and July 2020 through a Google forms’ online survey distributed on social media (Twitter, Facebook) and through a recruitment email sent to universities and healthcare centers. To be included in the study, participants had to be health professionals working in Quito at the time. Before completing the survey, a consent form was displayed on the first page. It contained information about the study’s aims as well as its risks and benefits. There were no incentives for participation; it was voluntary, anonymous, and people could withdraw from the study at any point. IRB approval was obtained by both universities involved before any data collection (Exp. num 143CER20 and cod. 20200414).

There was no pretest nor pilot study for this survey, which took approximately 15 min to be completed. A total of 117 healthcare professionals from three different fields of healthcare (physical, mental, and rehabilitation) working in Quito, Ecuador completed the anonymous online survey in Spanish with the following sections:

- Sociodemographic questionnaire. It included questions about participants’ age, gender, marital status, the field of healthcare, years as a healthcare professional and in the current job, the number of working hours and patients per week, percentage of high emotionally demanding cases per week, whether they had received additional training related to their professional activity in the last five years (yes/no), and whether they consider their salary to be adequate (yes/no).
- Professional Quality of Life questionnaire V [12] in the Spanish version [35]. It contains 30 items that measure CS, BO, and CF in formal caregivers. In this study, each subscale’s reliability was 0.80, 0.66, and 0.87, respectively. The answer options range from 1 (never) to 5 (always). Five items belonging to the BO subscale must be inverted. There is no total value; each construct has its score by adding the values of the 10 items of each subscale. The results of each subscale are interpreted as low, average, or high. This questionnaire has shown to have adequate psychometric properties in various cultural contexts [36].
- Work engagement [37]. This scale has nine items to measure three dimensions of work engagement. The items are scored on a scale ranging from 0 (not once) to 6 (every day). The score for each subscale is obtained by adding the items for each dimension and dividing the product by the number of items; the results range from 0 to 6 that are interpreted in each subscale as very low, low, average, high, and very high. In this study, Cronbach’s alpha was 0.87 for vigor, 0.92 for dedication, and 0.87 for absorption. In previous studies, it has shown good construct validity across samples [38].
- Scale of self-care behaviors for clinical psychologists [39]. This scale is originally in Spanish and has shown adequate internal consistency and construct validity. It has 10 items to quantify the frequency of self-care behaviors. The answer options range from 0 (never) to 4 (very frequently). There is a total score calculated by adding the responses. Higher scores translate to a higher frequency of these behaviors. The following categorization of frequency is used to better interpret the results according to the total score: 0 to 7 never, 8 to 15 almost never, 16 to 23 occasionally, 24 to 31 frequently, and 32 and above very frequently. The Cronbach’s alpha for this scale was 0.85.

All statistical analyses were conducted using SPSS version 25. We used descriptive statistics to characterize the sample and the overall responses. In addition, Pearson’s correlations, Analysis of Variance (ANOVA), and independent samples t-tests were used. Finally, linear regression analyses were carried out. All tests were two-tailed, and the significance level was set at *p* < 0.05. Only significant results are reported and, where percentages are shown, they are valid percentages. 

## 3. Results

A total of 120 professionals entered the survey. The analysis included subjects who completed at least 90% of it. A convenience sample of 117 healthcare professionals, working in Quito (Ecuador), was included in the study. The age range varied between 22 and 70 years (*M* = 40.65, *SD* = 12.56). Most participants (92.3%, n = 108) reported having had additional courses related to their professional activity in the last five years and only 38.8% (n = 45) considered their salary as adequate for their job.

Regarding professional experience, participants reported an average of 14.35 years (*SD* = 12.19) in their healthcare profession and 8.07 years (*SD* = 7.98) in their current job where, per week, they worked from 5 to 80 h (*M* = 33.84, *SD* = 19.05) and attended approximately 75 patients or family members (*SD* = 462). Out of the total number of weekly cases, participants report that on average, 44.0% are of high emotional demand for them (*SD* = 31.63). Table 1 summarizes the sociodemographic composition of the sample.

Participants reported high CS (*M* = 44.56, *SD* = 4.12), average CF (*M* = 24.83, *SD* = 7.4), and average BO (*M* = 24.17, *SD* =5.5). The results also show that people frequently engage in self-care practices (*M* = 24.48, *SD* = 6.98). Regarding engagement, the sample showed high levels of vigor (*M* = 4.81, *SD* = 1.11), dedication (*M* = 5.08, *SD* = 1.13), and absorption (*M* = 4.89, *SD* = 1.03).

Table 2 shows the correlations among the variables. Regarding the professional quality of life variables, CS positively related to age, self-care, vigor, dedication, and absorption while they were negatively correlated to BO. CF was positively associated with the number of patients per week and the high emotional demand they could represent. It also correlated positively with BO and negatively with self-care, vigor, dedication, and absorption. Lastly, BO is negatively related to all the engagement dimensions, age, number of years as a health professional, and CS. It is positively associated with the number of patients per week, the perception of not receiving an adequate salary, and CF. On the other hand, self-care is negatively related to the perception of not receiving an adequate salary, CF, and BO, positively related to CS, and the three dimensions of engagement. These dimensions also positively correlated with each other. In addition, vigor is positively related to age, number of years as a healthcare professional, and CS, and it is negatively correlated with the perception of not receiving an adequate salary, CF, and BO; finally, absorption and dedication are positively correlated with CS and negatively correlated with the perception of not receiving a fair salary, CF, and BO. 

To analyze the study variables’ differences (CS, CF, BO, self-care, vigor, dedication, and absorption) based on sociodemographic characteristics, we ran Student’s t-tests and ANOVAs. Table 3 shows the means and standard deviations of all the variables. There were no significant differences in the study variables by marital status, the number of working hours per week, the number of patients per week, nor the number of years in their current job. Moreover, there were no significant differences in CS subject to any of the variables. On the other hand, females showed higher CF scores than men, t(115) = 2.31, *p* = 0.02. According to the field of healthcare, there was also a significant difference in CF (*F*(2, 114) = 12.58, *p* < 0.001)). People working in emotional healthcare fields showed significant differences compared to those in physical and rehabilitation fields. CF scores also showed significant differences according to the percentage of emotionally demanding cases per week (*F*(2, 114) = 4.46, *p* = 0.01). People who attend a high percentage of emotionally demanding patients per week report higher CF.

In the case of BO, people showed different levels of it according to the number of years as a healthcare professional (*F*(3, 113) = 3.27, *p* = 0.02). Participants with less experience had higher levels of BO compared to those with more professional experience. In addition, people who perceived that they receive an adequate salary showed significantly less BO, t(114) = −2.15, *p* = 0.03.

Regarding self-care, people who received additional training in the last 5 years showed significantly higher self-care levels than those who had not, t(115) = 3.28, *p* = 0.001. Similarly, participants who perceived that they receive an adequate salary have more frequent self-care practices than those who do not, t(114) = 2.63, *p* = 0.01.

In the case of engagement, people had higher levels of vigor (t(115) = 3.33, *p* = 0.001), absorption (t(115) = 2.77, *p* = 0.006), and dedication (t(115) = 3.07, *p* = 0.003) when they had received additional training in the last 5 years. The same differences were observed for participants who perceived that they receive an adequate salary (vigor: t(115) = 2.36, *p* = 0.02; absorption: t(114) = 2.09, *p* = 0.04; and dedication: t(114) = 2.44, *p* = 0.02).

Finally, we tested which factors predict professional quality of life, self-care, and work engagement. Regression analyses were conducted with the variables that showed correlations and significant differences as predictors. For each model, first, we included all the variables mentioned above and then removed the non-significant ones. Significant models were found for CS (F(2, 114) = 30.41, *p* < 0.001, R^2^*_adj_* = 0.337), CF (F(3, 111) = 15.319, *p* < 0.001, R^2^*_adj_* = 0.274), BO (F(4, 110) = 25.861, *p* = 0.000, R^2^*_adj_* = 0.466), self-care (F(3, 113) = 30.468, *p* < 0.001, R^2^*_adj_* = 0.432), vigor (F(2, 114) = 206.334, *p* = 0.000, R^2^*_adj_* = 0.780), dedication (F(2, 114) = 204.617, *p* < 0.001, R^2^*_adj_* = 0.840), and absorption (F(1, 115) = 254.327, *p* < 0.001, R^2^*_adj_* = 0.686). Table 4 shows the standardized coefficients for each of the significant predictors of the models.

## 4. Discussion

This article explored the professional quality of life, work engagement, and self-care of healthcare professionals in Ecuador during the COVID-19 pandemic. Results indicate that the convenience sample that participated in the study presents average levels of CF and BO, frequent engagement in self-care practices, and high engagement and CS. 

### 4.1. Compassion Fatigue and Burnout

Accompanying patients and their relatives in their last days of life, in processes of disabling diseases, or during a global pandemic is not an easy task. The measures to mitigate the COVID-19 pandemic (e.g., confinement, increased responsibilities at home, economic instability, possible risk, or fear of contagion) can negatively influence the mental health [40] of people within the epidemiological fence [41] and in the general population [42]. Studies conducted during the first wave of COVID-19 indicate that the consequences of this stress can be so severe as to lead to suicidal thoughts [43].

As in another research [44], we found medium levels of CF and BO among the sample of professionals from Ecuador. There is currently no published research with the same variables to compare the obtained results in Ecuadorian samples. Most pre-pandemic studies in the country focused exclusively on BO. For example, a study carried out in 2017 indicated that 4.2% of health workers had high BO levels [45]. Another research from 2018 suggests that 20.7% of health professionals showed high emotional exhaustion levels [46]. In general, the present results could indicate an increase in BO, and thus a decrease in the professional quality of life, compared to previous data. However, this interpretation should be taken cautiously due to the different samples and questionnaires used. 

Our results contrast with the high levels of CF and BO perceived in previous studies with health professionals from Spain and Italy [33,34], which are two of the most affected countries in the European Union [47,48]. A possible explanation may be that the present study’s data collection was conducted over three months, in which the information about the development of the health crisis and the measures for its control were advancing. By contrast, the mentioned studies collected their data during the peak of the pandemic in Spain and Italy. Anyhow, the average CF and BO levels may indicate that the sample is at risk, so these results should be considered to propose intervention measures to reduce them.

Consistent with other research [33,34], female participants showed higher CF levels. This could be explained by the fact that during the time of data collection, women may have sustained a higher emotional and professional burden due to the confinement restrictions, online classes, and workload, given the high number of positive COVID-19 cases in Ecuador at the time. Our results also show that professionals in emotional health fields score the lowest on CF and BO, contrary to a previous study [49]. This suggests a different impact of the pandemic’s first wave on the health systems and their workforce. In other words, physical health was a priority, and thus, it may have had higher demands for its professionals at the time.

When analyzing the predictors of CF and BO, our regression results show that suffering from BO, being a woman, and caring for a high percentage of emotionally demanding patients could be factors that increase CF, as in a previous study from mental health professionals in Austria [50]. Our results also match those of Ruíz Fernandez et al. [44] where CS does not significantly influence CF. 

In the case of BO, we found that it is predicted by the number of weekly patients, the perception of an insufficient salary, CS, and suffering from CF. This last factor (CF) is also found in a previous study [44]. However, the perception of an insufficient salary as a predictor for BO is not consistent with other research, which found that BO was related to interest in work and organizational facilities but not with economic income [51]. This result might be due to the specific economic context in Ecuador.

### 4.2. Compassion Satisfaction, Engagement, and Self-Care

In the current study, the sample showed high CS levels, unlike other research carried out since the appearance of COVID-19 where moderate to high levels were shown [33,34]. It is possible that the very restrictive measures in Ecuador at the time of the study, which tried to stabilize the health system before any other areas, may have strengthened the already existing recognition of the healthcare task force. To accompany patients in a time of crisis, uncertainty, unwanted loneliness, and hospitalization without contact with their relatives may have also been a catalyst for the increase in CS [44]. 

Consistent with previous research [19], findings show a positive relationship between age and CS. The sample, adults (*M* = 40.65 years old) with an average of 14.35 years of professional experience, could have higher personal and professional maturity. In addition, previous experience as formal caregivers could have given them resources to better cope with complicated situations such as the current pandemic. When looking at the predictive model of CS, dedication was a significant variable and, as in Ruiz-Fernandez el at. [44], it also predicted CS, unlike age, which did not appear in our model.

Professionals with high engagement enjoy doing their job and feel that they are accomplished professionals [52]. There is a positive relationship between emotional demands and work engagement [53]. In fact, dedication is positively and significantly related to organizational commitment, job satisfaction, and enthusiasm [52]. In Ecuador, the disease’s incidence may have generated a labor force that is very engaged with their work, since very high levels of the three dimensions of engagement have been obtained: vigor, dedication, and absorption. These are similar to a study with Dutch intensivists and another one with Brazilian nurses who attended during the pandemic; both found high levels of engagement as well as its dimensions [54], especially in people over 40 years of age [55]. In addition, the positive work engagement that Ecuadorian health personnel shows as providers of care, and their high level of CS, may be related to the value of social networks and the promotion of social support as protective factors of psychological wellbeing [56].

Engagement also has the most predictive power to avoid BO [57]. All this contributes positively to wellbeing, job satisfaction, and job performance [58]. Our results also show high levels of total engagement negatively associated with BO. Regarding each of its components, we found that vigor’s predictive factors were self-care and dedication; those of dedication were CS, vigor, and absorption; and absorption was predicted by dedication. 

Regarding self-care, results indicate that the sample frequently engaged in such behaviors. The high levels of personal and organizational self-care (related to a perception of adequate salary and updated training in the last 5 years) may act as a buffer for CF and BO, as previous studies suggest [20,29,31]. Similarly, the high level of engagement and its relation to self-care practices agree with previous research that links them to the high levels of CS [32]. In terms of its predictor variables, the results show that having had additional training over the last five years, feeling vigor when doing one’s work, and having low levels of BO predict self-care practices.

## 5. Conclusions

The high levels of CS and engagement (vigor, dedication, and absorption) present a positively involved and committed workforce despite the difficulties surrounding COVID-19. Nonetheless, healthcare professionals and managers must be attentive to the average values obtained in CF and BO, which can be triggers for situations that could affect them and the quality of care they provide in the future. The practice of self-care behaviors is a strength. It promotes CS and engagement and is negatively related to CF, BO, and the perception of a low salary.

Given that this was a cross-sectional quantitative study, one limitation is its lack of longitudinal data to compare the same subjects’ results during the COVID-19 pandemic. In addition, due to the lockdown situation in Ecuador at the time of the study, we used a convenience sample recruited through social media and list servers. This affected the representativeness of the sample, and the interpretation of results must be carried out with caution. Despite this, the data obtained can lay the foundations for future studies to deepen the analysis of this situation’s impact on the professional quality of life, self-care, and engagement. Cautiousness should be also exerted when generalizing the results. Although one of the strengths is the sample size that highlights the participation of professionals who, despite working in extreme demands, offered their time to participate in the study, future studies could incorporate bigger samples and other cultural contexts. 

Despite the limitations, these findings contribute to understanding the predictors of professional quality of life, engagement, and self-care of healthcare professionals in Ecuador. These are of interest to formal caregivers, who can implement changes in their own life and people in management positions who can provide better support for their staff.

## Figures and Tables

**Table 1:** Sociodemographic characteristics of the sample.

Variable	*n*	*%*
Gender Male Female	43 74	36.75 63.25
Marital status Single Married Civil union Divorced Did not answer	45 42 7 22 1	38.5 35.9 6.0 18.8 0.9
Healthcare field Physical (medicine, nursing, nursing aid) Emotional (psychology and social work) Rehabilitation (physiotherapy and other)	74 23 20	63.25 19.66 17.09
Time as a healthcare professional 0 to 5 years 6 to 10 years 11 to 15 years More than 15 years	38 23 15 41	32.48 19.66 12.82 35.04
Time in current job 0 to 5 years 6 to 10 years 11 to 15 years More than 15 years	59 31 8 19	50.43 26.50 6.84 16.24
Working hours per week 0 to 20 20 to 40 More than 40	39 45 33	33.33 38.46 28.21
Number of patients per week 0 to 50 More than 50	95 22	81.20 18.80
% of emotionally demanding patients per week Low (0 to 30%) Medium (31 to 70%) High (71 to 100%)	52 38 27	44.44 32.48 23.08
Additional training in the last 5 years Yes No	108 9	92.31 7.69
Adequate salary (perceived) Yes No	45 71	38.46 60.68

**Table 2:** Pearson’s correlations among the study variables ^1,2^.

	M (*SD*)	1	2	3	4	5	6	7	8	9	10	11	12
1	40.65 (12.56)	-											
2	8.07 (7.98)	0.69 **	-										
3	14.35 (12.19)	0.93 **	0.70 **	-									
4	33.84 (19.05)	−0.06	−0.04	−0.08	-								
5	74.97 (462.27)	−0.06	−0.06	−0.07	0.03	-							
6	44.0 (31.63)	0.14	0.03	0.11	0.04	0.13	-						
7	44.56 (4.12)	0.22 *	0.09	0.14	−0.01	0.06	0.06	-					
8	24.83 (7.4)	−0.01	−0.04	0.01	0.10	0.19 *	0.24 **	−0.15	-				
9	24.17 (5.5)	−26 **	−0.18 *	−0.23 **	0.12	0.20 *	0.11	−0.55 **	0.45 **	-			
10	24.48 (6.98)	0.15	0.12	0.13	−0.13	−0.02	−0.03	0.41 **	−0.22 *	−0.53 **	-		
11	4.81 (1.11)	0.22 *	0.17	0.20 *	−0.03	−0.04	0.00	0.45 **	−0.32 **	−0.51 **	0.60 **	-	
12	5.08 (1.13)	0.17	0.15	0.15	−0.05	−0.06	−0.02	0.46 **	−0.32 **	−0.49 **	0.56 **	0.88 **	-
13	4.89 (1.03)	0.15	0.10	0.16	0.03	−0.17	0.02	0.28 **	−0.24 **	−0.32 **	0.46 **	0.75 **	0.83 **

^1^*Notes:* 1 = age, 2 = years in current job, 3 = years as a healthcare professional, 4 = working hours per week, 5 = number of patients per week, 6 = percentage of high emotional demand patients, 7 = CS, 8 = CF, 9 = BO, 10 = self-care, 11 = vigor, 12 = dedication, 13 = absorption. ^2^ * p < 0.05, ** *p* < 0.01, two-tailed.

**Table 3:** Mean and standard deviation for professional quality of life, self-care, and engagement by sociodemographic variables ^1,2^.

Variable		Professional Quality of Life			Self-Care	Engagement		
	*n*	CS *M (SD)*	CF *M (SD)*	BO *M (SD)*	*M (SD)*	VI ^1^ *M (SD)*	DE ^1^ *M (SD)*	AB ^1^ *M (SD)*
Gender Male Female	43 74	44.05 (4.34) 44.85 (3.99)	22.79 (6.22) * 26.01 (7.80) *	24.28 (6.04) 24.11 (5.21)	23.47 (7.65) 25.07 (6.54)	4.70 (1.28) 4.87 (1.01)	4.87 (1.35) 5.20 (0.97)	4.74 (1.15) 4.98 (0.95)
Marital status Single Married Civil union Divorced	45 42 7 22	44.09 (3.99) 44.55 (3.98) 41.86 (6.44) 46.14 (3.27)	25.24 (7.67) 24.52 (6.35) 23.29 (8.18) 23.91 (7.03)	25.29 (6.02) 23.29 (4.97) 24.14 (6.94) 23.32 (4.77)	24.96 (7.58) 24.67 (6.38) 21.29 (10.73) 4.91 (4.40)	4.68 (1.22) 5.01 (0.91) 4.24 (1.40) 5.03 (0.75)	4.99 (1.25) 5.25 (0.80) 4.62 (1.43) 5.24 (1.01)	4.81 (1.09) 5.09 (0.78) 4.57 (1.59) 4.94 (0.81)
Healthcare field Physical Emotional Rehabilitation	74 23 20	44.97 (3.85) 44.57 (4.39) 43.00 (4.62)	26.20 (7.14) ** 18.52 (4.29) ** 27.00 (7.55) **	24.08 (5.29) 23.13 (5.79) 25.70 (5.89)	24.64 (6.47) 25.04 (8.21) 23.25 (7.53)	4.83 (1.09) 4.93 (1.07) 4.57 (1.25)	5.09 (1.21) 5.23 (0.81) 4.83 (1.16)	4.90 (1.03) 5.01 (0.93) 4.72 (1.17)
Time as a healthcare professional 0 to 5 years 6 to 10 years 11 to 15 years More than 15 years	38 23 15 41	44.47 (3.53) 42.87 (5.42) 45.67 (4.30) 44.93 (3.48)	24.84 (8.73) 25.70 (7.15) 22.20 (6.99) 25.29 (6.32)	25.00 (5.35) * 26.48 (6.83) * 22.20 (5.54) * 22.83 (4.24) *	23.16 (7.61) 24.78 (8.49) 26.73 (6.25) 24.71 (5.55)	4.50 (1.30) 4.62 (1.21) 5.27 (0.94) 5.02 (0.82)	4.89 (1.33) 4.85 (1.28) 5.51 (0.71) 2.22 (0.93)	4.65 (1.35) 4.85 (1.04) 5.07 (0.57) 2.07 (0.77)
Time in current job 0 to 5 years 6 to 10 years 11 to 15 years More than 15 years	59 31 8 19	44.44 (4.50) 44.94 (3.70) 42.50 (4.07) 42.16 (3.53)	25.56 (8.70) 23.39 (5.40) 26.63 (5.99) 24.16 (6.26)	24.78 (6.08) 24.06 (5.33) 24.88 (3.76) 22.16 (4.15)	23.80 (7.32) 25.35 (7.46) 23.63 (5.95) 25.53 (5.53)	4.63 (1.24) 4.93 (1.05) 4.75 (0.94) 5.19 (0.76)	4.93 (1.32) 5.13 (1.01) 5.08 (0.56) 5.46 (0.79)	4.80 (1.24) 4.91 (0.77) 4.96 (0.45) 5.10 (0.87)
Working hours per week 0 to 20 20 to 40 More than 40	39 45 33	44.44 (3.86) 44.56 (4.44) 44.70 (4.08)	23.33 (6.58) 25.56 (7.85) 25.61 (7.64)	23.62 (5.54) 23.78 (5.55) 25.36 (5.37)	25.05 (7.62) 25.08 (7.02) 23.06 (6.08)	4.71 (1.05) 4.96 (1.06) 4.71 (1.26)	4.96 (1.11) 5.38 (0.93) 4.80 (1.33)	4.73 (1.11) 5.08 (0.91) 4.83 (1.08)
Number of patients per week 0 to 50 More than 50	95 22	44.48 (4.24) 44.86 (3.65)	24.45 (7.21) 26.45 (8.16)	24.29 (5.41) 23.64 (5.98)	24.14 (7.17) 25.95 (6.00)	4.79 (1.15) 4.88 (0.94)	5.06 (1.13) 5.14 (1.15)	4.88 (1.03) 4.91 (1.07)
% of emotionally demanding patients per week Low (0 to 30%) Medium (31 to 70%) High (71 to 100%)	52 38 27	44.44 (4.18) 44.71 (3.88) 44.56 (4.48)	23.06 (6.42) * 24.86 (6.64) * 28.15 (9.07) *	23.73 (5.73) 23.53 (4.58) 25.93 (6.05)	24.62 (7.58) 25.26 (6.13) 23.11 (6.96)	4.77 (1.18) 4.98 (0.87) 4.63 (1.27)	5.06 (1.12) 5.19 (1.00) 4.94 (1.34)	4.88 (1.02) 4.92 (1.03) 4.88 (1.11)
Additional training in the last 5 years Yes No	10 89	44.61 (3.96) 43.89 (6.03)	24.48 (6.80) 29 (12.41)	24.09 (5.41) 25.11 (6.79)	25.06 (6.71) ** 17.44 (6.67) **	4.90 (0.99) ** 3.67 (1.78) **	5.17 (1.01) ** 4.00 (1.90) **	4.97 (0.89) ** 4.00 (1.97) **
Adequate salary (perceived) Yes No	45 71	44.62 (3.66) 44.46 (4.42)	25.18 (7.21) 24.56 (7.60)	22.80 (4.56) * 25.03 (5.93) *	26.60 (2.30) * 23.17 (7.64) *	5.10 (0.61) * 4.61 (1.31) *	5.38 (0.64) * 4.87 (1.32) *	5.13 (0.69) * 4.73 (1.18) *

*Notes:*^1^ VI = vigor, DE = dedication, AB = absorption, ^2^ significant differences: * p < 0.05, ** *p* < 0.01, two-tailed.

**Table 4:** Linear regression models for professional quality of life (CS, CF, and BO), self-care, and work engagement (vigor, dedication, and absorption) ^1^.

	B	*SE*	*β*	*t*	*p*
CS					
(Constant)	47.57	2.76		17.27	<0.001
BO	−0.32	0.07	−0.42	−4.89	<0.001
Dedication	0.92	0.32	0.25	2.91	0.004
CF					
(Constant)	3.19	3.40		0.94	0.35
Gender	3.28	1.23	0.21	2.66	0.009
% of emotionally demanding patients	0.04	0.02	0.17	2.09	0.04
BO	0.61	0.11	0.45	5.56	<0.001
BO					
(Constant)	43.32	4.84		8.94	<0.001
Number of patients per week	0.002	0.001	0.15	2.06	0.04
Adequate salary (perceived)	2.12	0.77	0.19	2.75	0.007
CS	−0.66	0.10	−0.48	−6.92	<0.001
CF	0.26	0.05	0.36	5.01	<0.001
Self-care					
(Constant)	27.43	5.39		5.09	<0.001
Additional training in the last 5 years	−4.24	1.93	−0.16	−2.20	0.03
BO	−0.41	0.10	−0.32	−3.94	<0.001
Vigor	2.40	0.54	0.38	4.46	<0.001
Vigor					
(Constant)	0.26	0.23		1.14	0.257
Self-care	0.26	0.01	0.16	3.06	0.003
Dedication	0.77	0.05	0.79	14.99	<0.001
Dedication					
(Constant)	−1.04	0.48		−2.18	0.03
CS	0.03	0.01	0.11	2.71	0.008
Vigor	0.53	0.06	0.52	8.58	<0.001
Absorption	0.45	0.06	0.41	7.27	<0.001
Absorption					
(Constant)	1.06	0.45		4.28	<0.001
Dedication	0.76	0.05	0.83	15.95	<0.001

*Note:*^1^ Only significant predictors are shown.

## Data Availability

The data presented in this study are available on reasonable request from the corresponding author. The data are not publicly available due to ongoing analysis.
